# Muscle plays a more superior role than fat in bone homeostasis: A cross-sectional study of old Asian people

**DOI:** 10.3389/fendo.2022.990442

**Published:** 2023-01-12

**Authors:** Chaoran Liu, Pui Yan Wong, Xin Tong, Simon Kwoon-Ho Chow, Vivian Wing-Yin Hung, Wing-Hoi Cheung, Ling Qin, Sheung Wai Law, Ronald Man Yeung Wong

**Affiliations:** ^1^ Department of Orthopaedics & Traumatology, The Chinese University of Hong Kong, Hong Kong, Hong Kong SAR, China; ^2^ Department of Mechanical and Automation Engineering, The Chinese University of Hong Kong, Hong Kong, Hong Kong SAR, China; ^3^ Bone Quality and Health Centre, Department of Orthopaedics & Traumatology, The Chinese University of Hong Kong, Hong Kong, Hong Kong SAR, China

**Keywords:** osteoporosis, obesity, sarcopenia, muscle, fat

## Abstract

**Objectives:**

The aim of this study was to discover the role of fat and muscle in bone structures, as well as the relationship between obesity and sarcopenia on age-related osteoporosis.

**Methods:**

A total of 400 participants (65.0 ± 8.2 years old, 42.3% women) were recruited. Fat, muscle, bone parameters, basic demographics, medical history, physical performance and activity, and calcium intake of participants were obtained from datasets. The diagnosis of osteoporosis, sarcopenia, and obesity was based on current recommendations. Pearson correlation, non-linear regression models, and decision tree analyses were performed to study the relationship between fat, muscle, and bone. Logistic regression analyses were used to explore the risk of osteoporosis in old people with obesity or sarcopenia *via* Model 1 (unadjusted) and Model 2 (adjusted by age, physical activity, and calcium intake).

**Results:**

Correlation analysis showed that limb muscle mass and index, and age were best related to bone mineral density (BMD) (|*r|* = 0.386–0.632, *p* < 0.001). On the contrary, body mass index (BMI) and increased body fat percentage (BF%) were harmful for bone health. An increase of BMI and fat mass index slowed the increase of BMD in the spine, while skeletal muscle mass index accelerated the increase. People with sarcopenia had low muscle mass and strength. When separating subjects into sarcopenia and non-sarcopenia status, sarcopenia was independently related to higher risks of osteoporosis in both models (OR > 1, *p* < 0.05). BMI-defined obesity in Model 1 as well as BF%-defined obesity in both models did not reduce the risk of osteoporosis in both models (*p* > 0.05). The decision tree classification (85% accuracy) showed that greater body weight and larger lower limb muscle performance were negatively related to osteoporosis, while fat mass and percentage did not play roles in this prediction.

**Conclusion:**

Low muscle mass and function were harmful to bone health. Obesity defined by both BMI and BF% had limited protective roles in osteoporosis. The benefits for bone from increased muscle mass and function play a more superior role than increased fat mass in old people. Sarcopenia prevention and treatment instead of controlling obesity should be recommended as an approach to reduce the risks of age-related osteoporosis and fragility fracture for elderly people.

## 1 Introduction

Osteoporosis is characterized by low bone mineral density (BMD) and leads to a high risk of fracture in the elderly (1, 2). It is estimated that patients with fragility fractures will reach 6.3 million by 2050, posing a huge public health burden (3). The annual costs for fragility fracture patients will also increase 27% from 2017 to 2030 in Europe (4). In the United States (US), an average of US$3,000 per patient is required in the first year after a fracture (5).

The “obesity paradox” in osteoporosis has been reported previously, as people usually have a higher BMD due to increased mechanical loading (6, 7). In fact, studies have shown that for individuals with overweight or obesity, body mass index (BMI) reduces the risk of a fracture (8, 9). However, when body composition of fat and lean mass is taken into consideration, body fat mass is negatively associated with BMD in the younger population, while lean mass plays a positive role (10). In animal models, diet-induced obesity impairs bone turnover through cytokines and osteopontin secreted by adipose tissues, which activate osteoclasts and accelerate bone degradation (11). On the other hand, strain induced by muscle contraction stimulates bone growth as osteoblasts are mechanosensitive (6, 12). The skeletal muscle is one of the major components of lean mass. Muscle-secreted myokines including interleukins, irisin, myostatin, and growth factors can regulate bone metabolism (13). The composition of muscle and fat, as well as mechanical loadings, contributes to the bidirectional relationship of bone homeostasis.

The coexistence of osteoporosis and sarcopenia is prevalent in elderly people (14). Sarcopenia is an age-related muscle disorder, which is strongly related to fragility fractures in the elderly (15, 16). More importantly, it is currently unclear whether people with obesity have better bone microarchitecture compared to those with normal BMI after excluding individuals with sarcopenia. Dividing subjects into normal, obesity, sarcopenia, and sarcopenic obesity phenotypes can help to explore if the “obesity paradox” still exists in people with obesity when compared to healthy controls (17).

The aims of this study were to (1) investigate the correlation between muscle/fat and bone parameters (2); assess the osteoporosis risk according to normal, obesity, sarcopenia, and sarcopenic obesity groups; and (3) identify whether fat or muscle has a greater role in osteoporosis prediction.

## 2 Materials and methods

### 2.1 Study design and population

This study included the population aged 50 years or older from the established Normal Reference Study (NRS) cohort in May 2015 to April 2017. The aim of the cohort establishment was to study the reference centile curves of high-resolution peripheral quantitative computed tomography (HR-pQCT) parameters in Chinese adults in Hong Kong. The inclusion and exclusion criteria have been described previously (18). In brief, participants were physically healthy independent Chinese individuals, without medical conditions that affect bone metabolism, history of fragility fracture, or genetic skeletal disorders. Basic demographics, calcium intake, Baecke physical activity questionnaire, datasets of HR-pQCT, DXA, and medical history from each participant were obtained. The study procedure was approved by the local clinical research ethics committee (CRE.2014.310). All participants had written informed consent.

### 2.2 HR-pQCT assessment and image analysis

The distal radius and distal tibia (non-dominant and non-fractured) of participants were scanned by HR-pQCT (XtremeCT I, Scanco Medical AG, Switzerland) using the standard scanning protocol (60 kVp, 900 μA, 100 ms integration time, isotropic voxel size 82 μm). The details of the HR-pQCT operational procedures and the image quality grading of HR-pQCT for motion artifact were illustrated in previous studies (18, 19). Daily phantom calibration was performed to monitor the stability of the HR-pQCT system. Precision error was presented by coefficient of variation (CV). In the cohort, CV of vBMD ranged from 0.26% to 0.88%, and for microarchitectural parameters, it ranged from 0.56% to 3.78% (20). The proportion of good-quality image was 79.3% in the NRS cohort (18). Participants with poor-quality images including obvious image artifacts had been excluded in our study (21).

For image analysis, the volume of interest was differentiated into cortical and trabecular components using a fully automated cortical compartment segmentation technique (22). For bone density analysis, volumetric bone mineral density [vBMD, mg hydroxyapatite (HA)/cm^3^] for the whole bone, trabecular bone, and cortical bone were calculated. For trabecular bone analysis, bone volume fraction (BV/TV, %) was calculated from trabecular vBMD and the density of fully mineralized bone was assumed to be 1,200 mg HA/cm^3^ (23). Trabecular thickness (Tb.Th, mm) was derived by standard histomorphometry methods (24). For cortical bone analysis, cortical thickness was measured directly by a distance-transform method and intracortical pores in the binary cortex image were eliminated (19). Bone strength was estimated in terms of stiffness (kN/mm) by micro-finite element analyses (μFEA) on three-dimensional images of the distal radius and tibia using a built-in Image Processing Language software of HR-pQCT (IPL-FE v1.15, Scanco Medical). The specific conditions of the software operation were illustrated in previous studies (18, 19). Whole bone stiffness (kN/mm) was evaluated by performing a uniaxial compression test with 1000 N load and an apparent strain of 1%.

### 2.3 DXA, muscle function, and physical performance assessments

Body composition was measured by dual-energy x-ray absorptiometry (DXA) (Horizon; Hologic, Bedford, MA, USA). Scanning and analysis were performed under standard operating procedure by qualified technicians. Fat and lean mass (kg) in limbs, trunk, and whole body were measured and recorded. The ratio, percentage (divided by weight in kilograms), and index (normalized by height in meters squared) were calculated. Areal BMD (aBMD, g/cm^2^) at the proximal femur (femoral neck and total hip) and lumbar spine (L1 to L4) was evaluated. *T*-score and *Z*-score for total hip, lumbar spine, and femoral neck were evaluated. Daily phantom calibration was performed to monitor the stability of the DXA system. CV for femoral neck, total hip, and lumbar spine aBMD in our center was 1.36%, 1.19%, and 1.01%, respectively (19). Five elderly volunteers were included to assess the reliability of DXA examination. Briefly, they were asked to perform the examination at baseline and after 6 weeks, and not to participate in interventional research during this period. The intraclass correlation coefficient (ICC) with 95% CI of DXA-measured limb lean mass, total fat mass, aBMD of femoral neck, total hip, and lumbar spine was 0.915 (0.507, 0.991), 0.988 (0.888, 0.999), 0.972 (0.790, 0.997), 0.943 (0.546, 0.994), 0.992 (0.726, 0.999) (*p*≤0.004) .

Handgrip strength (HGS) was measured by the Smedley dynamometer (model EH101, Camry). Participants were in standing position with elbows in full extension and grasped the dynamometer as hard as they could for three times on both hands (30-s rest between each trial) (25). The highest reading among all trials was taken for analysis. Lower limb strength was also measured by the dynamometer (model EH101, Camry). The test aimed to examine the isometric strength of the quadriceps and hip extensors predominately (26). Participants were in sitting position on a highchair with both legs hung loose. The ankle was tied with the straps that connect with the dynamometer with knee angle at 90°C flexion. The participants then raised the leg forward as hard as they could for three times on both legs (30-s rest between each trial). The highest reading was taken for analysis. Physical performance was assessed by gait speed (GS). Participants walked a total distance of 8 m at usual speed and the time for walking the middle 6-m distance was measured by a manual stopwatch. The GS (m/s) was calculated as the distance (6 m) divided by the average time of the two trials for analysis. Five elderly volunteers were also included to assess the reliability of these tests. The reliability of HGS, lower limb strength, and GS was 0.985 (0.883, 0.998), 0.942 (0.575, 0.994), and 0.990 (0.916, 0.999) (*p* ≤ 0.003).

### 2.4 Diagnosis of osteoporosis, sarcopenia, and obesity

With reference to the *T*-score, BMD was defined as osteoporosis when *T*-score ≤ −2.5 in either femoral neck, total hip, or total lumbar spine (27). To diagnose sarcopenia, the Asian Working Group for Sarcopenia (AWGS) 2019 criteria were used (28). Those with low muscle mass and low HGS/GS were identified as sarcopenia. DXA-measured appendicular skeletal muscle mass index (ASMI) <7.0 kg/m^2^ in male subjects and <5.4 kg/m^2^ in female subjects were regarded as low muscle mass. Low muscle strength was indicated by HGS <28 kg in men and <18 kg in women, and low physical performance was indicated by GS <1 m/s. A recent consensus recommends using BF% to define obesity, which not only regarded the body composition assessment but was shown to be more suitable for elderly people (29). Therefore, two obesity definitions were utilized, BMI ≥ 25 kg/m^2^ and BF% > 60th percentile of the current cohort (male subjects > 32.7%, female subjects > 43.2%) for assessment (29–31). Those with the presence of sarcopenia and obesity were defined as “sarcopenic obesity (SO)”.

### 2.5 Calcium intake and physical activity assessments

Daily calcium intake was measured by a validated local food frequency questionnaire (32). Participants were asked to report the frequency and usual consumption amount of each food item in the past year by a trained interviewer; memory recall of the food consumption pattern was aided by photo booklet and different portion reference tools. The total calcium content from the reported food was calculated for analysis. The habitual physical activity over the previous 12 months was assessed by the modified Baecke questionnaire (33). Several questions in each of four categories (work, sport, leisure time, and housework) were asked with score ranging from 1 (never) to 5 (always or often). The total score from the four categories was calculated for the analysis of physical activity assessment.

### 2.6 Statistical analysis

The consistency and stability of DXA, muscle function, and performance were analyzed by ICC. To study the correlation between muscle/fat and bone structure at different sites, we performed Pearson correlation coefficient test and demonstrated a heatmap according to fat and muscle indicators, as well as bone parameters. Muscle indicators included physical activity and performance. Bone parameters were from the hip and spine by DXA, and distal radius and tibia by HR-pQCT. The block color closer to white represented nonsignificance. The correlation coefficient (*r*) of 0–0.19, 0.2–0.39, 0.4–0.59, 0.6–0.79, and 0.8–1.0 was regarded as very weak, weak, moderate, strong, and very strong correlation, respectively. Nonparametric regression models were executed to display the bone parameter in different bone sites with increasing BMI, ASMI, or fat mass index (FMI). One-way ANOVAs with Bonferroni tests were performed to compare the muscle, fat, and bone parameters of normal, obese, sarcopenic, and SO groups. Continuous variables were exhibited as mean and standard deviation (SD). Odds ratio (OR) of osteoporosis, osteopenia, lower radius and tibial vBMD of participants with obesity, sarcopenia, and SO were performed with logistic regression. Normal people were the reference group (OR = 1.00). Briefly, the logistic regression model was chosen, and the diagnosis of osteoporosis was the outcome variable. In Model 1 (unadjusted), only sarcopenia/obesity/SO and normal status were covariates. In Model 2 (adjusted), age, physical activity score, and calcium intake were also considered as covariates. The odds ratio was read from EXP(B) with 95% CI. Low vBMD was defined as lower than 2 SD of the local young reference (20 to 35 years old) mean value. According to the 240 young subjects from the NRS cohort, the cutoffs of lower radial vBMD were 268.7 mg HA/cm^3^ in male subjects and 267.4 mg HA/cm^3^ in female subjects, and for tibia, they were 230.5 mg HA/cm^3^ in male subjects and 248.9 mg HA/cm^3^ in female subjects. We used a machine learning method (decision tree classification) to predict osteoporosis. Basic demographics, muscle and fat indicators, calcium intake, and fall and medical history were the selected attributes. The ratio of training sets and test sets was 7:3 (34). The specificity, sensitivity, and accuracy of the classification were detected. Python 3.10.1, R 4.0.2, and SPSS Statistics 20 were utilized for all analyses and visualizations. Significant difference was defined as *p* < 0.05.

## 3 Results

A total of 400 subjects (42.3% of which were female) from 50 to 84 (mean ± SD: 65.0 ± 8.2) years old were included. Thirteen subjects had missed at least one of the physical performance or activity data, and 38 subjects missed the calcium intake calculation. These participants were not included in the odds ratio and decision tree analyses. All participants had DXA and HR-pQCT parameters.

### 3.1 The effects of fat and muscle parameters on different sites of bone

**Figure 1:**
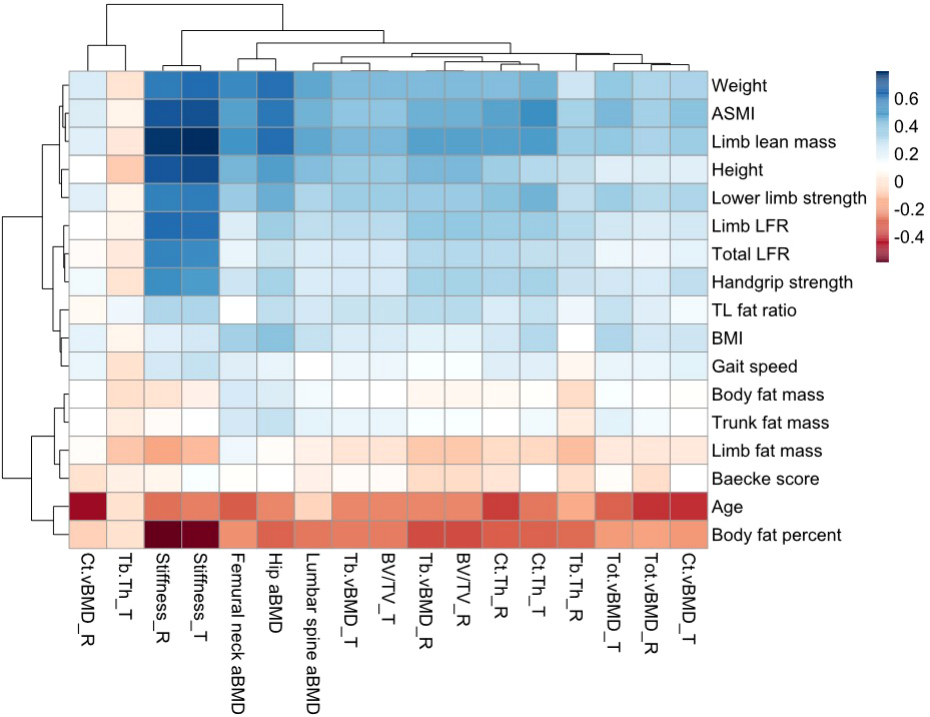
Correlation heatmap of muscle, fat, and bone indicators. The color intensity of the block represented the strong or weak correlation between two parameters. Blue blocks showed the positive correlation (*r* = 0–0.8), while the red showed the negative correlation (*r* = −0.8–0). The color closer to white could be regarded as no statistical significance (*p* > 0.05). TL, trunk-to-limb; LFR, lean-to-fat ratio; T, tibia; R, radius.

The correlation heatmap is shown in **Figure 1**. For annotations of all parameters, please refer to **Supplementary Table S1**. Increased age was negatively correlated with bone homeostasis, displayed by the lower BMD at the sites of hip, lumbar spine, tibia, and radius, as well as BV/TV and cortical bone thickness in both tibia and radius, and trabecular bone thickness in radius. The radial cortical bone vBMD was the most relevant parameter, which showed moderately negative correlation with age (*r* = −0.473, *p* < 0.001). BMI was positively correlated to various bone parameters, particularly the weight-bearing hip aBMD (*r* = 0.400, *p* < 0.001), but it showed no correlation with trabecular bone thickness in both radius and tibia (*p* > 0.05). Another obesity-related indicator was body fat percentage (BF%). The majority of the correlation coefficient in BF% was opposite to BMI findings. The stiffness of the tibia and radius was reduced with higher BF% (*r* ≤ −0.563, *p* < 0.001). Weak negative correlations were found in BMD and BF% in the tibia, hip, and lumbar spine (*r* = −0.297 to −0.193, *p* < 0.001), but very weak in radius (*r* = −0.176, *p* < 0.001). The absolute body fat mass was positively associated with hip, lumbar spine, and tibial BMD instead of radius. Considering fat distribution, we observed that limb fat mass was only correlated with femoral neck aBMD, but not BMD in other sites (*r* = 0.165, *p* = 0.001). Radius and tibial stiffness and trabecular bone thickness as two fracture predictors were negatively correlated with limb fat mass (35). Conversely, trunk fat mass was positively associated with BMD in all sites, as well as trabecular bone thickness and tibial stiffness (*p* < 0.05). Trunk-to-limb fat ratio showed positive associations with most bone parameters except for cortical bone vBMD in radius.

Limb muscle mass and ASMI were weakly associated with radial vBMD (*r* = 0.335, *p* < 0.001) and strongly associated with total hip aBMD (*r* = 0.605–0.632, *p* < 0.001). Both muscle indicators showed obviously positive correlations with bone parameters, especially the stiffness of peripheral bone (*r* = 0.685–0.803, *p* < 0.001). However, they were not associated with tibial trabecular bone thickness (*p* > 0.05). The lean-to-fat ratio in limb and total body had similar results. Additionally, the functional performance was positively correlated with most bone parameters. Limb muscle mass and index had the strongest positive correlation parameters for radial vBMD, and age had the largest negative correlation (*p* < 0.001). ASMI was most positively related to tibial vBMD, while age and BF% were negatively related to it (*p* < 0.001). For both total hip and lumbar spine aBMD, limb lean mass has the strongest positive correlation coefficient and BF% had the strongest negative one (*p* < 0.001).

### 3.2 The increase rate of BMD with rising BMI, ASMI, and FMI

**Figure 2:**
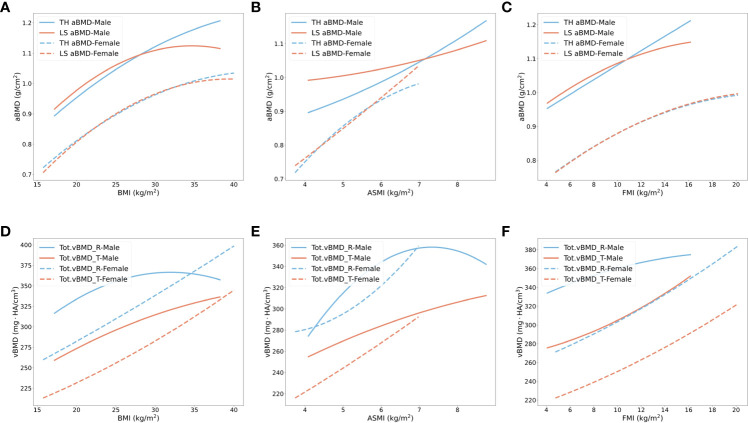
Non-linear regression models of BMD in total hip, lumbar spine, radius, and tibia with the increased body weight, muscle, and fat index. **(A–C)** Total hip (blue) and lumbar spine (orange) aBMD changes with BMI, ASMI, and FMI in male subjects (solid line) and female subjects (dashed line). **(D–F)** Radius (blue) and tibias (orange) vBMD changes with BMI, ASMI, and FMI in male subjects (solid line) and female subjects (dashed line). TH, total hip; LS, lumbar spine; R, radius; T, tibia.

It has been reported that BMI and fracture risk have a non-linear relationship (36). We used second-order non-linear regression models to explore the relationships between BMI, ASMI (eliminate influence of organs in trunk), FMI, and BMD in different bone sites, shown in **Figure 2**. With increasing BMI, a slower increase rate of BMD in male subjects at all bone sites and female subjects at hip and lumbar spine was found when BMI was over approximately 25 kg/m^2^ (quadratic coefficient < 0). However, the rising trend of radial and tibial BMD in female subjects did not change significantly (**Figures 2A, D**). To observe whether the role of different body compositions in BMD was similar to BMI, we used ASMI as muscle index, and FMI as fat index. An increase in ASMI accelerated BMD enhancement in hip and spine in male subjects, and spine in female subjects (quadratic coefficient > 0) (**Figures 2B, E**). However, when BMI and ASMI increased to a certain threshold (31.9 kg/m^2^ and 7.3 kg/m^2^), radius BMD started to decrease in male subjects. Consistent with BMI, as FMI increases, there is also a slower rate of increase for hip and spine BMD in both genders, as well as radius BMD in male subjects (quadratic coefficient < 0) (**Figures 2C, F**). Increasing FMI benefits the tibial BMD increment in both genders (quadratic coefficient > 0).

### 3.3 Osteoporosis risk according to sarcopenic and obese status

We separated the older participants into normal, obese, sarcopenic, and sarcopenic obese groups according to sarcopenia and obesity definitions. **Tables 1**, **2** show the bone parameters in each group when obesity was defined by BMI and BF%, respectively.

**Table 1:** Muscle, fat, and bone parameters in normal, obese, sarcopenic, and SO groups when obesity was defined by BMI.

Group	Male				Female			
	Normal	Obesity	Sarcopenia	SO	Normal	Obesity	Sarcopenia	SO
***N***	108	70	43	10	71	42	43	13
**Age (years)**	63.06 ± 8.06^a^	62.06 ± 8.91^a^	70 ± 6.77^b^	71.1 ± 8.57^b^	64.79 ± 7.06^ab^	62.19 ± 7.11^b^	67.88 ± 7.3^a^	69.77 ± 5.95^a^
**BMI (kg/m^2^)**	22.73 ± 1.59^a^	27.96 ± 2.6^b^	22.36 ± 1.93^a^	26.73 ± 1.67^b^	22.19 ± 1.97^a^	28.37 ± 2.94^b^	21.49 ± 2.31^a^	26.15 ± 1^c^
**Weight (kg)**	64.55 ± 5.92^a^	77.47 ± 7.2^b^	61.27 ± 6.34^c^	71.04 ± 8.53^d^	53.55 ± 5.59^ac^	67.55 ± 9.15^b^	50.29 ± 5.99^a^	57.86 ± 4.45^c^
**Height (cm)**	168.45 ± 5.31^a^	166.54 ± 5.4^ab^	165.51 ± 5.58^b^	162.78 ± 6.54^b^	155.31 ± 4.91^a^	154.14 ± 5.53^a^	152.98 ± 5.43^ab^	148.7 ± 5.44^b^
**Body fat (kg)**	19 ± 3.1^a^	26.76 ± 4.49^b^	18.94 ± 3.27^a^	25.38 ± 5.09^b^	20.69 ± 3.55^a^	30.33 ± 5.82^b^	20.47 ± 4.08^a^	26.46 ± 2.18^c^
**Body fat percentage (%)**	29.89 ± 3.39^a^	34.94 ± 3.33^b^	31.42 ± 3.44^a^	36.2 ± 3.69^b^	39.06 ± 3.9^a^	45.32 ± 3.68^b^	41.08 ± 4.23^c^	46.47 ± 2.33^b^
**ASMI (kg/m^2^)**	6.55 ± 0.57^a^	7.46 ± 0.54^b^	6.14 ± 0.63^c^	6.66 ± 0.32^ac^	5.23 ± 0.48^a^	5.94 ± 0.49^b^	4.72 ± 0.42^c^	5.06 ± 0.28^ac^
**Limb fat mass (kg)**	7.78 ± 1.33^a^	10.29 ± 1.67^b^	7.62 ± 1.24^a^	10.05 ± 2.05^b^	9.34 ± 1.92^a^	13.5 ± 3.03^b^	9.01 ± 1.68^a^	11.2 ± 1.29^c^
**Trunk fat mass (kg)**	9.92 ± 2.02^a^	15.03 ± 3^b^	10.06 ± 2.24^a^	13.99 ± 3.13^b^	10.22 ± 2.01^a^	15.61 ± 3.38^b^	10.36 ± 2.67^a^	14.09 ± 1.69^b^
**Limb lean mass (kg)**	18.6 ± 2.04^a^	20.58 ± 1.86^b^	16.84 ± 1.96^c^	17.68 ± 1.63^ac^	12.63 ± 1.4^a^	14.11 ± 1.68^b^	11.04 ± 1.14^c^	11.22 ± 1.2^c^
**TL fat ratio**	1.28 ± 0.2^a^	1.47 ± 0.19^b^	1.32 ± 0.23^a^	1.4 ± 0.15^ab^	1.12 ± 0.22^a^	1.18 ± 0.21^a^	1.15 ± 0.21^a^	1.27 ± 0.21^a^
**Handgrip strength (kg)**	35.65 ± 5.17^a^	36.07 ± 5.74^a^	28.65 ± 5.49^b^	30.86 ± 4.41^b^	23.75 ± 17.58^a^	21.54 ± 4.23^a^	18.16 ± 3.65^a^	16.39 ± 3.34^a^
**Lower limb strength (kg)**	31 ± 8.35^a^	32.28 ± 7.54^a^	25.6 ± 6.67^b^	27 ± 6.96^ab^	19.94 ± 5.41^a^	21.17 ± 9.05^a^	15.52 ± 4.43^b^	13.69 ± 4.42^b^
**Gait speed (m/s)**	1.23 ± 0.17^a^	1.18 ± 0.24^a^	0.91 ± 0.24^b^	0.94 ± 0.21^b^	1.15 ± 0.21^a^	1.12 ± 0.25^a^	0.94 ± 0.19^b^	0.85 ± 0.24^b^
**Baecke score**	8.15 ± 1.42^a^	7.86 ± 1.34^a^	7.74 ± 1.4^a^	7.66 ± 1.39^a^	7.93 ± 1.05^a^	7.78 ± 1.25^a^	7.55 ± 1.16^a^	7.26 ± 1.14^a^
**Femoral neck aBMD (g/cm^2^)**	0.76 ± 0.11^a^	0.82 ± 0.13^b^	0.7 ± 0.09^c^	0.78 ± 0.07^abc^	0.67 ± 0.12^a^	0.73 ± 0.11^b^	0.61 ± 0.09^c^	0.64 ± 0.12^ac^
**Total hip aBMD (g/cm^2^)**	1.02 ± 0.12^a^	1.1 ± 0.13^b^	0.94 ± 0.11^c^	1.06 ± 0.09^ab^	0.88 ± 0.12^a^	0.94 ± 0.11^b^	0.81 ± 0.1^c^	0.87 ± 0.09^abc^
**Lumbar spine aBMD (g/cm^2^)**	1.03 ± 0.16^a^	1.08 ± 0.19^a^	1 ± 0.2^a^	1.14 ± 0.16^a^	0.86 ± 0.16^a^	0.96 ± 0.16^b^	0.81 ± 0.13^a^	0.86 ± 0.12^ab^
**Femoral neck *T*-score**	−0.69 ± 0.71^a^	−0.3 ± 0.84^b^	−1.11 ± 0.63^c^	−0.55 ± 0.49^abc^	−1 ± 1.3^a^	−0.37 ± 1.17^b^	−1.74 ± 0.98^c^	−1.41 ± 1.33^ac^
**Femoral neck *Z*-score**	0.43 ± 0.93^a^	0.91 ± 1.07^b^	0.15 ± 0.86^a^	0.96 ± 0.52^ab^	0.5 ± 1.12^ab^	0.94 ± 1.02^a^	0 ± 0.88^b^	0.47 ± 1.15^ab^
**Total hip *T*-score**	0.12 ± 0.86^a^	0.73 ± 0.88^b^	−0.45 ± 0.8^c^	0.39 ± 0.67^ab^	−0.1 ± 1.14^a^	0.49 ± 1^b^	−0.71 ± 0.96^c^	−0.13 ± 0.87^abc^
**Total hip *Z*-score**	1.09 ± 0.94^ac^	1.67 ± 0.96^b^	0.74 ± 0.8^c^	1.79 ± 0.59^ab^	1.12 ± 1.08^ab^	1.59 ± 0.93^a^	0.65 ± 0.89^b^	1.33 ± 0.69^ab^
**Lumbar spine *T*-score**	0.25 ± 1.53^a^	0.8 ± 1.81^a^	0.01 ± 1.9^a^	1.32 ± 1.49^a^	−1.08 ± 1.58^a^	−0.27 ± 1.48^b^	−1.63 ± 1.26^a^	−1.17 ± 1.04^ab^
**Lumbar spine *Z*-score**	0.54 ± 1.06^a^	0.97 ± 1.24^a^	0.38 ± 1.21^a^	1.3 ± 1.02^a^	0.46 ± 1.15^a^	1.03 ± 1.11^b^	0.23 ± 0.94^a^	0.62 ± 0.81^ab^
Radius								
**Tot.vBMD (mg HA/cm^3^)**	350.16 ± 63.28^ab^	368.07 ± 65.38^a^	325.95 ± 56.76^b^	350.89 ± 56.49^ab^	306.1 ± 74.42^ab^	330.59 ± 78.87^a^	274.83 ± 63.13^b^	297.21 ± 68.36^ab^
**Ct.vBMD (mg HA/cm^3^)**	889.23 ± 51.16^a^	891.3 ± 48.33^a^	856.12 ± 67.34^b^	879.97 ± 56.62^ab^	863.43 ± 68.37^ab^	890.99 ± 64.09^a^	847.52 ± 60.86^b^	862.1 ± 55.43^ab^
**Ct.Th (mm)**	0.96 ± 0.19^a^	1.01 ± 0.2^a^	0.86 ± 0.22^b^	0.96 ± 0.19^ab^	0.75 ± 0.19^ab^	0.84 ± 0.18^a^	0.68 ± 0.17^b^	0.71 ± 0.16^ab^
**Tb.vBMD (mg HA/cm^3^)**	156.54 ± 33.53^ab^	169.15 ± 36.96^a^	147.84 ± 23.86^b^	152.81 ± 23.26^ab^	120.43 ± 36.08^a^	124.94 ± 37.51^a^	104.98 ± 32.57^a^	110.14 ± 33.14^a^
**BV/TV (%)**	0.13 ± 0.03^ab^	0.14 ± 0.03^a^	0.12 ± 0.02^b^	0.13 ± 0.02^ab^	0.1 ± 0.03^a^	0.1 ± 0.03^a^	0.09 ± 0.03^a^	0.09 ± 0.03^a^
**Tb.Th (mm)**	0.08 ± 0.01^a^	0.08 ± 0.01^a^	0.08 ± 0.01^a^	0.08 ± 0.01^a^	0.07 ± 0.01^a^	0.07 ± 0.01^a^	0.07 ± 0.01^a^	0.08 ± 0.02^a^
**Stiffness (kN/mm)**	91.82 ± 15.34^a^	96.59 ± 15.78^a^	83.09 ± 15.2^b^	86.78 ± 14.11^ab^	58.67 ± 11.13^ac^	62.88 ± 10.06^a^	51 ± 8.43^b^	51.08 ± 7.07^bc^
Tibia								
**Tot.vBMD (mg HA/cm^3^)**	289.56 ± 45.76^a^	309.1 ± 48.04^b^	272.07 ± 43.6^a^	302.11 ± 58.43^ab^	251.87 ± 55.67^a^	272.86 ± 55.06^a^	219.73 ± 38.86^b^	274.07 ± 35.58^a^
**Ct.vBMD (mg HA/cm^3^)**	846.96 ± 44.52^a^	856.25 ± 45.78^a^	817.31 ± 52.67^b^	842.1 ± 60.66^ab^	796.43 ± 63.93^a^	832.34 ± 65.65^b^	777.19 ± 55.62^a^	807.11 ± 42.33^ab^
**Ct.Th (mm)**	1.24 ± 0.23^a^	1.34 ± 0.26^b^	1.14 ± 0.26^a^	1.37 ± 0.32^ab^	0.95 ± 0.26^a^	1.07 ± 0.26^a^	0.81 ± 0.2^b^	1.04 ± 0.21^a^
**Tb.vBMD (mg HA/cm^3^)**	160.65 ± 27.48^ab^	168.71 ± 28.36^a^	151.48 ± 27.93^b^	155.05 ± 27.36^ab^	138.15 ± 33.08^a^	142.35 ± 33.42^a^	119.83 ± 28.57^b^	140.85 ± 21.17^ab^
**BV/TV (%)**	0.13 ± 0.02^ab^	0.14 ± 0.02^a^	0.13 ± 0.02^b^	0.13 ± 0.02^ab^	0.12 ± 0.03^a^	0.12 ± 0.03^a^	0.1 ± 0.02^b^	0.12 ± 0.02^ab^
**Tb.Th (mm)**	0.08 ± 0.01^a^	0.08 ± 0.01^a^	0.08 ± 0.01^a^	0.08 ± 0.01^a^	0.08 ± 0.01^ab^	0.08 ± 0.01^ab^	0.08 ± 0.01^a^	0.09 ± 0.02^b^
**Stiffness (kN/mm)**	224.1 ± 29.91^a^	233.48 ± 31.25^a^	205.72 ± 30.92^b^	217.72 ± 30.96^ab^	155.76 ± 25.87^a^	167.75 ± 23.47^b^	135 ± 18.62^c^	149.17 ± 14.83^abc^

Significant differences: different letters (a, b, c, or/and d), p < 0.05. One-way ANOVA followed by Bonferroni test. LFR, lean-to-fat ratio; TL, trunk-to-limb.

**Table 2:** Muscle, fat, and bone parameters in normal, obese, sarcopenic, and SO groups when obesity was defined by BF%.

Group	Male				Female			
	Normal	Obesity	Sarcopenia	SO	Normal	Obesity	Sarcopenia	SO
***N***	106	72	30	23	70	43	30	26
**Age (years)**	61.75 ± 4.24^a^	64.01 ± 4.95^a^	70.07 ± 12.02^b^	70.39 ± 4.95^b^	64.44 ± 11.31^ab^	62.81 ± 6.08^a^	68.4 ± 7.62^b^	68.23 ± 6.36^ab^
**BMI (kg/m^2^)**	23.19 ± 8.18^ab^	27.14 ± 12.63^c^	22.26 ± 5.95^a^	24.38 ± 8.36^b^	22.64 ± 10.02^a^	27.5 ± 3.6^b^	20.84 ± 2.15^c^	24.57 ± 2.23^d^
**Weight (kg)**	65.63 ± 17.96^a^	75.52 ± 32.53^b^	60.74 ± 18.81^c^	66.21 ± 19.52^ac^	54.25 ± 24.18^a^	66.1 ± 9.43^b^	48.53 ± 5.39^c^	56.11 ± 5.2^a^
**Height (cm)**	168.23 ± 6.86^a^	166.91 ± 2.47^ab^	165.24 ± 3.25^ab^	164.67 ± 3.11^b^	154.79 ± 3.54^a^	155.01 ± 4.93^a^	152.61 ± 5.07^ab^	151.26 ± 6.33^b^
**Body fat (kg)**	18.81 ± 8.23^a^	26.83 ± 14.11^b^	17.69 ± 6.82^a^	23.38 ± 7.37^c^	20.6 ± 13.96^a^	30.26 ± 5.56^b^	18.77 ± 3.24^a^	25.43 ± 2.78^c^
**Body fat percentage (%)**	29.11 ± 4.94^a^	35.95 ± 4.81^b^	29.62 ± 2.23^a^	35.84 ± 0.7^b^	38.41 ± 8.97^a^	46.23 ± 2.49^b^	39.14 ± 3.34^a^	46.02 ± 2.23^b^
**ASMI (kg/m^2^)**	6.79 ± 2.14^a^	7.07 ± 1.83^b^	6.28 ± 1.35^c^	6.19 ± 2.04^c^	5.4 ± 1.71^a^	5.66 ± 0.6^a^	4.76 ± 0.43^b^	4.85 ± 0.41^b^
**Limb fat mass (kg)**	7.67 ± 3.92^a^	10.37 ± 4.53^b^	7.16 ± 1.94^a^	9.27 ± 3.15^c^	9.14 ± 6.68^a^	13.72 ± 2.64^b^	8.34 ± 1.33^a^	10.87 ± 1.36^c^
**Trunk fat mass (kg)**	9.82 ± 4.26^a^	15.04 ± 9.24^b^	9.25 ± 4.62^a^	12.81 ± 3.98^c^	10.3 ± 7.07^a^	15.35 ± 3.45^b^	9.35 ± 2.11^a^	13.4 ± 2.15^c^
**Limb lean mass (kg)**	19.02 ± 4.54^a^	18.77 ± 4.45^a^	17.14 ± 4.38^b^	16.81 ± 4.81^b^	12.94 ± 3.95^a^	13.24 ± 2.67^a^	11.08 ± 1.15^b^	11.09 ± 1.15^b^
**TL fat ratio**	1.29 ± 0.09^a^	1.46 ± 0.16^b^	1.3 ± 0.29^a^	1.39 ± 0.05^ab^	1.15 ± 0.12^a^	1.13 ± 0.19^a^	1.12 ± 0.2^a^	1.24 ± 0.22^a^
**Handgrip strength (kg)**	36.41 ± 6.29^a^	34.95 ± 0.71^a^	30.17 ± 7.78^b^	27.58 ± 7.07^b^	23.88 ± 3.82^a^	21.37 ± 3.66^a^	18.64 ± 3.97^a^	16.75 ± 2.98^a^
**Lower limb strength (kg)**	31.71 ± 0^a^	31.2 ± 0^ac^	26.83 ± 10.61^bc^	24.55 ± 11.31^b^	20.33 ± 2.12^a^	20.5 ± 8.33^a^	15.28 ± 4.97^b^	14.88 ± 3.89^b^
**Gait speed (m/s)**	1.22 ± 0.15^a^	1.2 ± 0.23^a^	0.95 ± 0.05^b^	0.86 ± 0.03^b^	1.18 ± 0^a^	1.06 ± 0.22^b^	0.92 ± 0.19^c^	0.92 ± 0.22^c^
**Baecke score**	8.32 ± 0.19^a^	7.63 ± 1.3^b^	7.83 ± 2.45^ab^	7.58 ± 0.38^ab^	7.96 ± 0.62^a^	7.75 ± 1.24^a^	7.63 ± 1.17^a^	7.31 ± 1.13^a^
**Femoral neck aBMD (g/cm^2^)**	0.77 ± 0.01^ab^	0.8 ± 0.22^a^	0.72 ± 0.2^b^	0.71 ± 0.08^b^	0.69 ± 0.19^a^	0.7 ± 0.09^a^	0.6 ± 0.09^b^	0.64 ± 0.1^ab^
**Total hip aBMD (g/cm^2^)**	1.03 ± 0.05^ac^	1.08 ± 0.27^a^	0.95 ± 0.3^b^	0.98 ± 0.11^bc^	0.89 ± 0.27^a^	0.91 ± 0.11^a^	0.8 ± 0.1^b^	0.85 ± 0.1^ab^
**Lumbar spine aBMD (g/cm^2^)**	1.03 ± 0.06^a^	1.08 ± 0.17^a^	1.02 ± 0.33^a^	1.03 ± 0.05^a^	0.89 ± 0.23^a^	0.91 ± 0.15^a^	0.8 ± 0.13^b^	0.85 ± 0.13^ab^
**Femoral neck *T*-score**	−0.61 ± 0.07^ab^	−0.42 ± 1.41^a^	−0.99 ± 1.34^b^	−1.02 ± 0.49^b^	−0.82 ± 2.05^a^	−0.68 ± 0.99^a^	−1.87 ± 1.01^b^	−1.42 ± 1.1^ab^
**Femoral neck *Z*-score**	0.47 ± 0.07^a^	0.85 ± 1.63^a^	0.28 ± 1.34^a^	0.28 ± 0.92^a^	0.65 ± 1.2^a^	0.68 ± 0.87^a^	−0.1 ± 0.77^b^	0.35 ± 1.1^ab^
**Total hip *T*-score**	0.2 ± 0.42^a^	0.6 ± 1.98^b^	−0.37 ± 2.19^c^	−0.2 ± 0.78^ac^	0.07 ± 2.55^a^	0.19 ± 1^a^	−0.75 ± 1.01^b^	−0.38 ± 0.9^ab^
**Total hip *Z*-score**	1.13 ± 0.57^a^	1.61 ± 1.84^b^	0.81 ± 1.84^a^	1.04 ± 1.06^ab^	1.28 ± 2.05^a^	1.32 ± 0.97^a^	0.64 ± 0.8^b^	1.01 ± 0.96^ab^
**Lumbar spine *T*-score**	0.24 ± 0.49^a^	0.79 ± 1.7^a^	0.23 ± 3.11^a^	0.3 ± 0.49^a^	−0.83 ± 2.12^a^	−0.69 ± 1.39^a^	−1.76 ± 1.22^b^	−1.25 ± 1.18^ab^
**Lumbar spine *Z*-score**	0.54 ± 0.28^a^	0.95 ± 1.27^a^	0.52 ± 2.05^a^	0.55 ± 0.28^a^	0.61 ± 1.06^a^	0.77 ± 1.05^a^	0.14 ± 0.85^a^	0.52 ± 0.96^a^
Radius								
**Tot.vBMD (mg HA/cm^3^)**	350.89 ± 66.4^ab^	366.51 ± 17.11^a^	338. ± 123.04^ab^	320.0 ± 156.13^b^	318.34 ± 160.3^a^	310.0 ± 74.79^ab^	268.04 ± 66.71^b^	293.86 ± 60.03^ab^
**Ct.vBMD (mg HA/cm^3^)**	891.52 ± 10.54^a^	887.86 ± 35.43^a^	870.6 ± 83.93^ab^	847.5 ± 175.15^b^	873.3 ± 120.14^a^	874.19 ± 60.09^a^	841.3 ± 62.56^a^	861.98 ± 54.8^a^
**Ct.Th (mm)**	0.97 ± 0.01^a^	0.99 ± 0.06^a^	0.91 ± 0.45^ab^	0.84 ± 0.6^b^	0.79 ± 0.37^a^	0.78 ± 0.18^a^	0.66 ± 0.17^b^	0.72 ± 0.16^ab^
**Tb.vBMD (mg HA/cm^3^)**	155.96 ± 73.75^a^	169.66 ± 56.07^b^	152.5 ± 33.73^ab^	143.92 ± 23.62^a^	124.45 ± 37.19^a^	118.29 ± 36.48^a^	104.7 ± 34.58^a^	107.89 ± 30.46^a^
**BV/TV (%)**	0.13 ± 0.06^a^	0.14 ± 0.05^b^	0.13 ± 0.03^ab^	0.12 ± 0.02^a^	0.1 ± 0.03^a^	0.1 ± 0.03^a^	0.09 ± 0.03^a^	0.09 ± 0.03^a^
**Tb.Th (mm)**	0.08 ± 0.02^a^	0.08 ± 0^a^	0.08 ± 0.01^a^	0.08 ± 0^a^	0.07 ± 0.02^a^	0.07 ± 0.01^a^	0.07 ± 0.01^a^	0.07 ± 0.02^a^
**Stiffness (kN/mm)**	93.53 ± 15.73^a^	93.94 ± 17.49^a^	86.68 ± 30.97^ab^	80.02 ± 30.07^b^	60.37 ± 13.91^a^	60.02 ± 10.57^a^	50.81 ± 9.24^b^	51.25 ± 6.67^b^
Tibia								
**Tot.vBMD (mg HA/cm^3^)**	290.6 ± 11.38^ab^	306.91 ± 84.85^a^	277.13 ± 126.64^b^	278.54 ± 76.93^ab^	260.83 ± 68.31^a^	257.8 ± 52.89^a^	218.64 ± 41.36^b^	248.15 ± 43.06^ab^
**Ct.vBMD (mg HA/cm^3^)**	851.23 ± 8.56^a^	849.7 ± 65.41^a^	825.8 ± 84.92^ab^	816.9 ± 119.15^b^	807.48 ± 114.41^a^	813.52 ± 63.46^a^	775.47 ± 58.25^a^	794.13 ± 47.81^a^
**Ct.Th (mm)**	1.26 ± 0.21^a^	1.32 ± 0.25^a^	1.18 ± 0.76^a^	1.18 ± 0.54^a^	1.01 ± 0.39^a^	0.98 ± 0.26^a^	0.81 ± 0.2^b^	0.93 ± 0.23^ab^
**Tb.vBMD (mg HA/cm^3^)**	159.9 ± 19.73^ab^	169.58 ± 63.99^a^	152.85 ± 49^b^	151.25 ± 1.56^b^	139.12 ± 20.15^a^	140.67 ± 34.54^a^	118.82 ± 30.73^b^	131.52 ± 24.03^ab^
**BV/TV (%)**	0.13 ± 0.02^ab^	0.14 ± 0.05^a^	0.13 ± 0.04^b^	0.13 ± 0^b^	0.12 ± 0.02^a^	0.12 ± 0.03^a^	0.1 ± 0.03^b^	0.11 ± 0.02^ab^
**Tb.Th (mm)**	0.08 ± 0.01^a^	0.08 ± 0^a^	0.08 ± 0^a^	0.08 ± 0.01^a^	0.08 ± 0^a^	0.08 ± 0.01^a^	0.08 ± 0.02^a^	0.08 ± 0.02^a^
**Stiffness (kN/mm)**	227.15 ± 13.42^a^	228.73 ± 41.97^a^	209.01 ± 67.59^b^	206.65 ± 32.28^b^	158.84 ± 40.96^a^	162.46 ± 25.01^a^	134.78 ± 19.71^b^	142.34 ± 16.9^b^

Significant differences: different letters (a, b, c, or/and d), p < 0.05. One-way ANOVA followed by Bonferroni test. LFR, lean-to-fat ratio; TL, trunk-to-limb.

When obesity was defined by BMI (**Table 1**), hip aBMD was lowest in people with sarcopenia and highest in people with obesity in both genders (*p* < 0.05). Male individuals with SO had higher total hip aBMD than individuals with sarcopenia, but female individuals with SO had a similar value compared to the other three groups. Lumbar spine aBMD significantly increased in female subjects with obesity than in normal individuals with sarcopenia, but male subjects had comparable values among the four groups. Poor muscle status did not contribute to the BMD at this site (*p* > 0.05). Obese and normal groups had similar radial vBMD, and people with sarcopenia had lower values than individuals with obesity (*p* < 0.05). Similarly, other radius parameters were comparable between obese and normal groups, and most of the parameters were remarkedly decreased in people with sarcopenia compared to those with obesity. In male tibia, the total distal tibial vBMD and cortical bone thickness were increased in individuals with obesity, and sarcopenic and normal groups had similar values. Nevertheless, female subjects had higher tibial cortical bone vBMD in the obese compared to normal groups, but not the total vBMD. Female subjects with sarcopenia had lowest tibial vBMD, cortical bone thickness, and BV/TV than both normal and obese groups. Radial and tibial stiffness values significantly decreased in people with sarcopenia (*p* < 0.05). Higher stiffness in the obese group was only found in female tibia compared to the normal group. Older individuals with sarcopenic obesity generally had comparable bone parameters compared to other groups. When obesity was defined by BF% (**Table 2**), male subjects with obesity had increased ASMI, which was not found in female subjects. Limb lean mass and BMD in all bone sites were similar between obese and normal groups (*p* > 0.05). Compared to the normal group, the sarcopenic group had lower hip aBMD. Decreased spine, radius, and tibia BMD were only found in female subjects with sarcopenia, while male subjects with SO had reduced radius vBMD (*p* < 0.05).

To detect the effects of sarcopenia and obesity on impaired bone homeostasis, ORs with 95% confidence interval (CI) of osteoporosis, low radius and tibial vBMD, and fall history according to the above grouping are shown in **Tables 3** and **4** based on different obesity definitions. Before the model adjustment, BMI-defined obesity was associated with lower risks of osteoporosis and low tibial vBMD (OR < 1.0, *p* < 0.05), but not low radial vBMD. Sarcopenia was associated with higher risks of osteoporosis, low radius and tibial vBMD, and falls (OR > 1.0, *p* < 0.05). After adjusted by age, physical activity, and calcium intake, obesity did not significantly affect bone status, while sarcopenia was still independently related to higher risks of osteoporosis, low tibial vBMD, and falls. Individuals with sarcopenic obesity had similar risks of bone impairments and fall compared to normal people. When obesity was defined by BF%, sarcopenia was associated with lower BMD in all bone sites, and sarcopenic obesity was related to higher fall risk (OR > 1.0, *p* < 0.05). However, obesity was not significantly associated with reduced osteoporosis risks (*p* > 0.05). In the adjusted model, the role of sarcopenia as a risk factor in low radial vBMD was no longer present.

**Table 3:** The odds ratio of osteoporosis, low radial and tibial vBMD, and fall of obesity, sarcopenia, and SO compared to normal groups when obesity was defined by BMI.

Group		Normal	Obesity	Sarcopenia	SO
Model 1					
Osteoporosis	OR (95% CI)	1.00 (reference)	0.262 (0.075, 0.917)^a^	3.079 (1.527, 6.207)^a^	1.429 (0.385, 5.310)
	*p*		0.036	0.002	0.594
Low radial vBMD	OR (95% CI)	1.00 (reference)	0.551 (0.271, 1.122)	1.991 (1.094, 3.621)^a^	1.621 (0.593, 4.435)
	*p*		0.100	0.024	0.347
Low tibial vBMD	OR (95% CI)	1.00 (reference)	0.538 (0.294, 0.984)^a^	2.681 (1.565, 4.592)^a^	0.421 (0.120, 1.483)
	*p*		0.044	<0.001	0.178
Fall	OR (95% CI)	1.00 (reference)	1.251 (0.583, 2.687)	3.079 (1.527, 6.207)^a^	2.647 (0.873, 8.030)
	*p*		0.565	0.002	0.086
Model 2					
Osteoporosis	OR (95% CI)	1.00 (reference)	0.370 (0.100, 1.367)	2.925 (1.319, 6.483)^a^	1.241 (0.285, 5.400)
	*p*		0.136	0.008	0.774
Low radial vBMD	OR (95% CI)	1.00 (reference)	0.738 (0.347, 1.56)	1.609 (0.800, 3.236)	1.704 (0.544, 5.342)
	*p*		0.430	0.182	0.361
Low tibial vBMD	OR (95% CI)	1.00 (reference)	0.581 (0.297, 1.136)	2.771 (1.498, 5.120)^a^	0.361 (0.093, 1.405)
	*p*		0.112	0.001	0.142
Fall	OR (95% CI)	1.00 (reference)	1.234 (0.537, 2.837)	2.581 (1.103, 6.040)^a^	3.303 (0.874, 12.478)
	*p*		0.621	0.029	0.078

a Statistical significance, p < 0.05. Model 1: unadjusted; Model 2: adjusted by age, physical activity, and calcium intake.

**Table 4:** The odds ratio of osteoporosis, low radial and tibial vBMD, and fall of obesity, sarcopenia, and SO compared to normal groups when obesity was defined by BF%.

Group		Normal	Obesity	Sarcopenia	SO
Model 1					
Osteoporosis	OR (95% CI)	1.00 (reference)	0.360 (0.117, 1.107)	3.953 (1.847, 8.464)^a^	1.667 (0.644, 4.314)
	*p*		0.064	<0.001	0.292
Low radial vBMD	OR (95% CI)	1.00 (reference)	0.675 (0.341, 1.336)	2.086 (1.059, 4.107)^a^	1.947 (0.935, 4.055)
	*p*		0.257	0.033	0.075
Low tibial vBMD	OR (95% CI)	1.00 (reference)	0.732 (0.410, 1.304)	3.093 (1.678, 5.703)^a^	1.365 (0.679, 2.743)
	*p*		0.288	<0.001	0.383
Fall	OR (95% CI)	1.00 (reference)	0.874 (0.400, 1.912)	2.066 (0.936, 4.560)	3.305 (1.513, 7.221) ^a^
	*p*		0.736	0.072	0.003
Model 2					
Osteoporosis	OR (95% CI)	1.00 (reference)	0.451 (0.135, 1.505)	3.469 (1.420, 8.476)^a^	1.306 (0.447, 3.820)
	*p*		0.195	0.006	0.626
Low radial vBMD	OR (95% CI)	1.00 (reference)	0.706 (0.333, 1.497)	1.357 (0.602, 3.058)	1.449 (0.611, 3.436)
	*p*		0.364	0.461	0.400
Low tibial vBMD	OR (95% CI)	1.00 (reference)	0.653 (0.341, 1.253)	2.649 (1.291, 5.433)^a^	1.372 (0.622, 3.024)
	*p*		0.200	0.008	0.433
Fall	OR (95% CI)	1.00 (reference)	1.180 (0.512, 2.719)	1.579 (0.570, 4.377)	3.402 (1.294, 8.938)^a^
	*p*		0.698	0.380	0.013

a Statistical significance, p < 0.05. Model 1: unadjusted; Model 2: adjusted by age, physical activity, and calcium intake.

### 3.4 Contribution of fat and muscle indicators in osteoporosis identification

**Figure 3:**
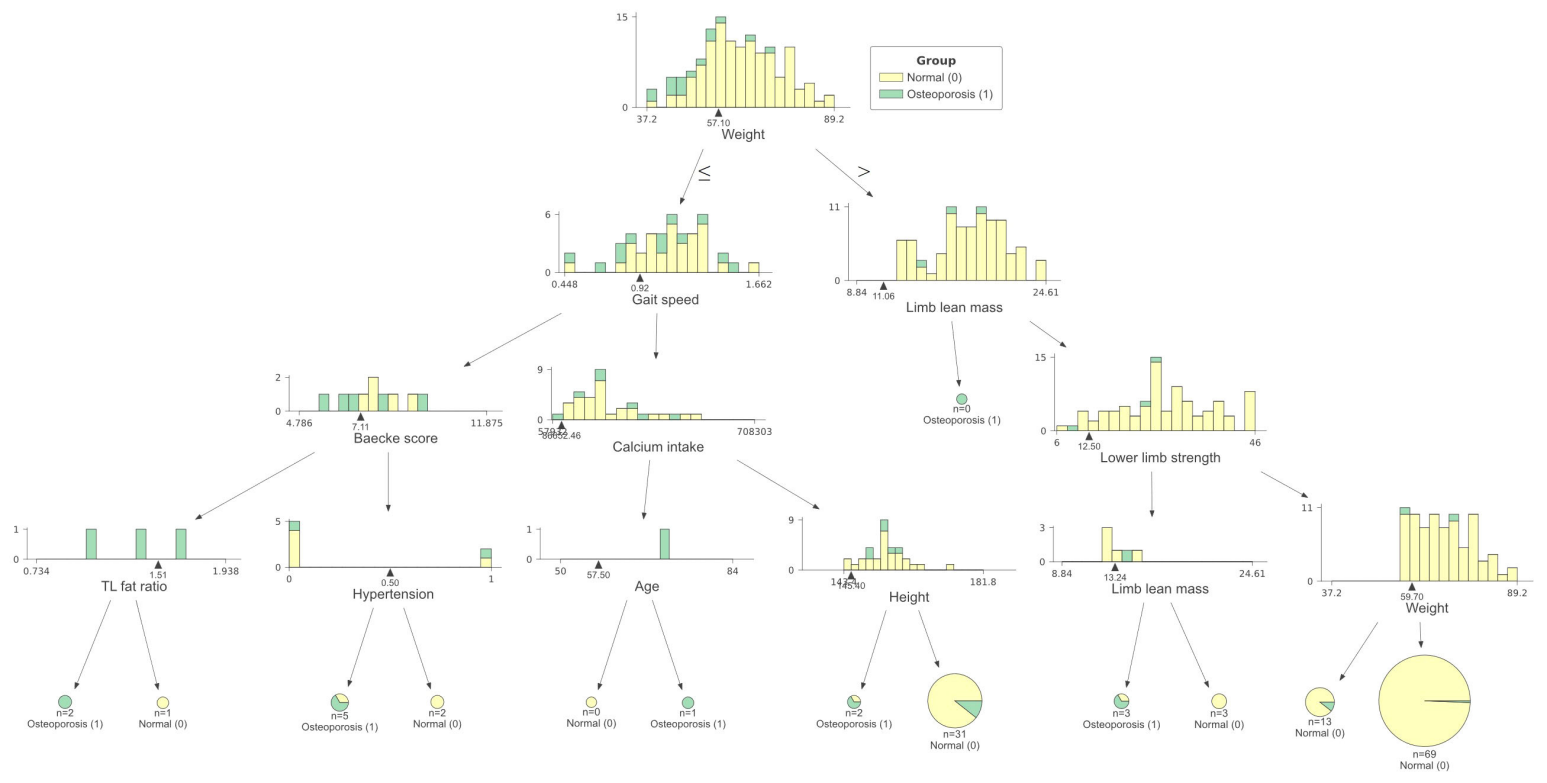
Machine learning decision tree classification of age-related osteoporosis according to basic information, muscle and fat indicators, physical activity, calcium intake, and medical history. Testing showed that the decision tree with a depth of 4 had a specificity of 0.92, a sensitivity of 0.27, and an accuracy of 0.85 to classify osteoporosis individuals. Normal = 0 (yellow), Osteoporosis = 1 (green).

We conducted the established “white box” decision tree machine learning model to detect osteoporosis based on the weight, height, age, BMI, muscle and fat indicators, medical history (hypertension, diabetes mellitus, dyslipidemia, cardiovascular diseases, fatty liver, autoimmune diseases, and malignancy), calcium intake, Baecke score, smoking, and drinking status. A four-depth model was chosen since the increased depths did not obviously improve the accuracy and may lead to overfit (**Figure 3**). According to the test, the most significant node (root) was body weight, followed by gait speed and limb lean mass. Baecke score, calcium intake, lower limb strength, trunk-to-limb fat ratio, hypertension status, age, and height were also selected. The decision tree had a specificity of 0.92, a sensitivity of 0.27, and an accuracy of 0.85. The prevalence osteoporosis in people with body weight lower than 57.1 kg was higher (27.3%) compared to those with higher weight (3.4%). People with both lower weight and gait speed (<0.92 m/s) had higher prevalence of osteoporosis (50.0%) compared to those with lower weight and higher gait speed (20.6%). Old people with higher weight but reduced lower limb strength (<12.5 kg) had higher prevalence of osteoporosis (16.7%) than those with higher lower limb strength (2.4%). Old people with lower body weight and gait speed had higher prevalence of osteoporosis. For those with a higher body weight and increased lower limb strength, the prevalence of osteoporosis was lowest.

## 4 Discussion

Increased weight, limb lean mass, ASMI, physical performance, and lean-to-fat ratio were significantly associated with better bone health, while increased BF%, age, and limb fat mass were harmful. BMI as an indicator for obesity definition was not as relevant to bone as the above parameters. Age-related BMD reduction was more obvious in non-weight-bearing bone radius than other bone sites. The body fat mass was not as stronger related to bone parameter as muscle mass. It is reported that BF% can better predict clinical outcomes as compared to BMI in the elderly population, which is still applicable in bone turnover research (37). High BF% and limb fat mass dramatically impairs bone strength, which indicates that fat infiltration in the bone marrow may occur (38). Our results showed that trunk-to-limb fat ratio mildly benefit bone structure, since these populations usually have higher body weight and bone mechanical stimulation. However, abdominal obesity was related to increased hip fracture risk after normalized by BMI (39). The trunk fat mass measured by DXA included not only abdominal fat, but also fat in the pelvis area; therefore, our data could not show the impact of abdominal obesity on bone health. The skeletal muscle has positive roles in bones, as it regulates metabolism and provides mechanical stimulations (loading and straining) (6, 12, 40). Muscle-related indicators including functions were significantly associated with bone parameters. Lower limb strength was better correlated with bone parameters than HGS and gait speed. Total hip BMD was strongly related to limb lean mass, which shows the cause of hip fracture frequently occurring in patients with sarcopenia (16). Stiffness as a predictor of frailty fracture increased along with muscle mass and functional performance (35). BMD as the diagnostic index for osteoporosis was more positively relevant to muscle than fat mass and BMI, and negatively related with age and BF% in both weight-bearing and non-weight-bearing bones. Herein, muscle mass and function had more benefits in BMD and bone stiffness than fat mass.

Similar to the relationship between BMI and fracture risk, BMI and BMD had a non-linear relationship in hip and spine in both genders, and peripheral bones in male subjects only (36). These findings indicate that with BMI increment, the increase rate of BMD gradually flattened out. The radius BMD in male subjects started to decrease after the BMI reached 31.9 kg/m^2^. Since the radius is a non-weight-bearing bone, excessive weight may impair bone homeostasis, but only plays minor mechanical roles (41). Different from BMI and FMI, an increase in ASMI accelerated BMD enhancement in hip and spine in male subjects and spine in female subjects, indicating that a higher ASMI is beneficial to bone homeostasis. The changing trends of FMI and BMD were more similar to BMI with the comparison of ASMI. In female subjects, the deposit of fat was not as adverse as in male subjects. It may be attributed to the sex differences of fat distribution, as pear-shaped body fat distribution in female subjects bring fewer metabolic problems than the central obesity in male subjects (42). In summary, we found from the non-linear models that with an increase in BMI, the rate of increase for BMD generally slows down, except for tibia and radius in female subjects. Fat and muscle mass indexes play different roles in the increase rate. The balance of bone mechanical stimulation and unhealthy metabolic status tilts to the latter with the BMI increment. In non-weight-bearing bones, the downtrend of BMD was found in male subjects with obesity. Excessive muscle instead of fat increment did not slow down the increase rate of BMD in hip and spine.

There is a disease classification for old people that can reflect the muscle and fat compositions (43). Obesity alone, sarcopenia alone, sarcopenic obesity, and normal groups are used to study the importance of muscle and fat on bone. BMI is a commonly used indicator to define obesity. After removing the patients with sarcopenia from the normal BMI population, individuals with BMI-defined obesity only had limited bone parameters better than the healthy control group, including total hip BMD in both genders and tibial BMD in male subjects. People with sarcopenia had lower total hip BMD, as well as radial and tibial stiffness than the normal individuals. Poor muscle status played significantly negative roles in diverse bone sites except for spine, while obese status only slightly improved bone structure in hip. Hence, people with normal BMI and good muscle status have similar bone microstructure and strength compared to people with obesity. Old people with SO had similar bone parameters compared to other groups, indicating that fat prevented negative effects from the loss of muscle mass, and poor muscle status attenuated the protective effects of fat mass. Considering body composition, those with higher BMI might have higher muscle mass instead of fat mass, but are regarded as obese. Increased body fat mass was more related to clinical outcomes in old individuals than BMI (44, 45). Therefore, BF% is recommended to identify obesity in old people. When obesity was defined by BF%, BMD was comparable between obese and normal groups, which indicated that BF%-defined obesity did not reduce the risk of osteoporosis as expected from the “obesity paradox”. Without considering muscle mass, the increased fat mass percentage in old people did not benefit BMD. Although BMI-defined obesity is related to lower risks of osteoporosis in bearing bones, the protective role did not exist after the adjustments of age, physical activity, and calcium intake. However, sarcopenia is still a dependent risk factor of osteoporosis. The lower muscle mass had stronger effects on the development of osteoporosis. BF%-defined obesity was not significantly related to reduced osteoporosis risks in both models. SO instead of sarcopenia was associated with fall risks when obesity was defined by BF%. Since non-weight-bearing bones were less related to obesity and sarcopenia, the prevention of fall might be the best approach to reduce the fracture risk of those sites. From the odds ratio of fall, the prevention or treatment of sarcopenia and SO should be performed for old people to avoid frailty fracture of non-weight-bearing bones. Patients with sarcopenia hiding in the normal BMI population were the confounding factor that created a false impression of the “obesity paradox”; therefore, BF% might be a better indicator for obesity diagnosis in old people. From the current results, the “obesity paradox” did not exist in old people with obesity defined by BF%, which was consistent with basic science studies and underlying molecular mechanisms (11). From the odds ratio results, sarcopenia is more associated with age-related osteoporosis than obesity, which emphasized the role of muscle in osteoporosis (46).

From the decision tree testing, we observed that the sensitivity of osteoporosis classification according to the involved parameters was low. The underlying reason is that this cohort only recruited those with a low risk of abnormal bone metabolism. Numerous known influencing factors of osteoporosis have already been excluded. Our study compared the role of muscle and fat in age-related bone health. Few confounding factors related to secondary osteoporosis were involved in this study. We could still extract information from the current model, since the accuracy is 85%. Body weight is the most important predictor of osteoporosis. Lower limb performances were also good indicators to predict osteoporosis. Those with greater weight and higher lower limb strength had lower risks of osteoporosis. Higher gait speed can protect people with low weight from osteoporosis compared to those with lower gait speed. Higher limb lean mass also slightly contributed to osteoporosis prevention in the prediction model. Fat mass as well as BMI were not automatically included. Muscle and bone are physically close to each other, and our findings demonstrated that they also have a functional crosstalk. It provides implications for clinicians and nutritionists that body weight, muscle function, and mass for pre-onset osteoporosis patients should be focused on, because skeletal muscle not only provides more mechanical stimulation, but also beneficially affects both bone and systemic metabolism.

**Figure 4:**
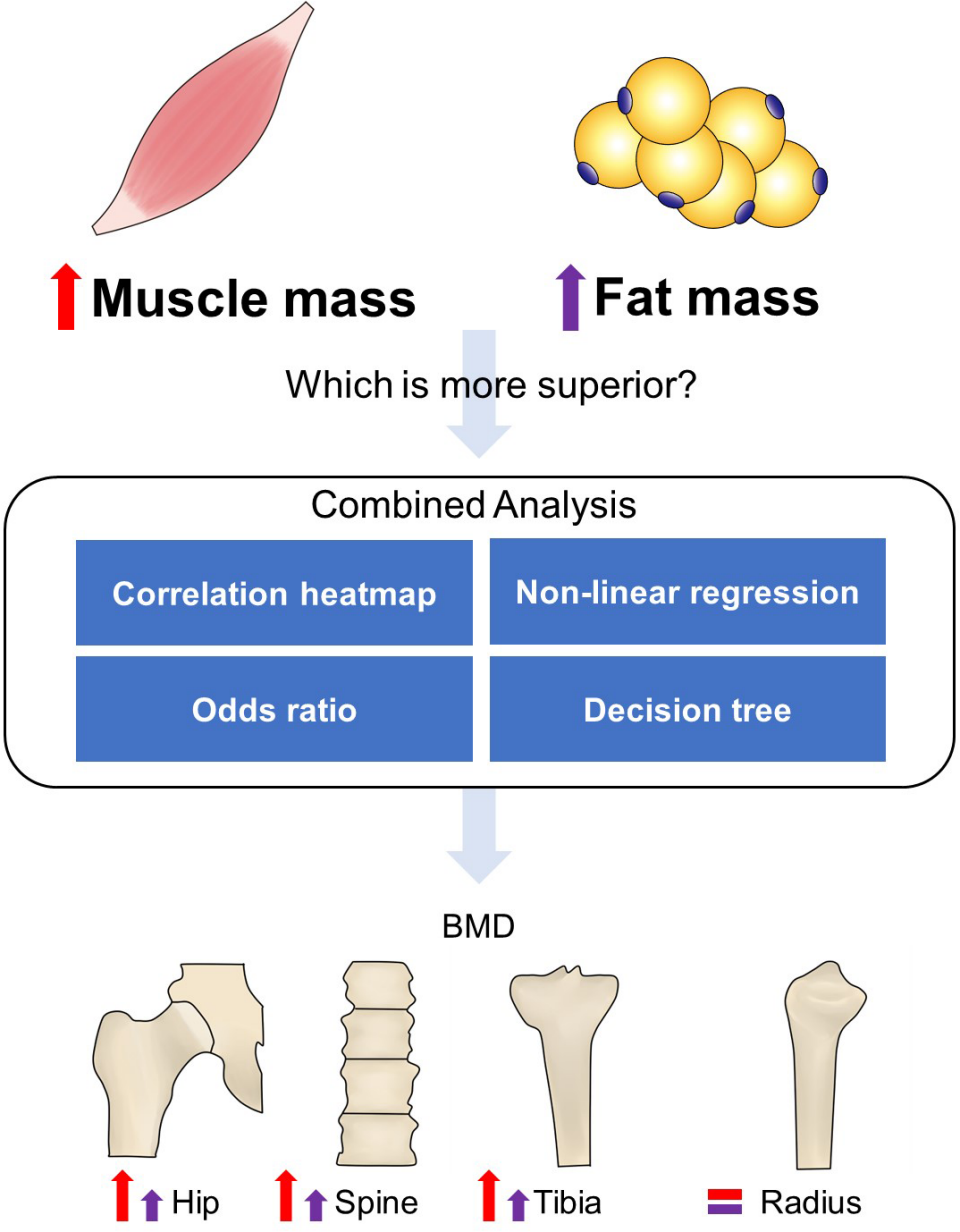
Flowchart of the study design. A combined analysis was performed to compare the role of muscle and fat in bone homeostasis. Correlation heatmap showed that the increased muscle mass-related indexes had a stronger positive correlation with bone structure compared to fat mass-related indexes. Non-linear regression showed that the increase rate of BMD in hip and spine almost did not slow down with increased ASMI but FMI. The odds ratio with 95% CI indicated that sarcopenia induced high incidence of osteoporosis, while obesity did not effectively prevent it compared to the normal group. The decision tree also showed that greater weight, muscle performance, and limb lean mass can be negative predictors of osteoporosis. The red and purple up arrows indicated more remarkable roles of increased muscle mass in weight-bearing bones’ (hip, spine, and tibia) health than fat mass, while radius was not significantly affected by them.

There are several strengths to our study. To our knowledge, this is the first research to study the muscle and fat effects on bone health together and compare their contributions (**Figure 4**). We indicated that obesity did not prevent osteoporosis when compared to people with normal BMI or BF% and without sarcopenia. The bone sites with or without weight bearing had different reflections on BMI, muscle, and fat alterations, as well as gender. Our study also had limitations. Our cohort excluded those with disease-induced osteoporosis, and thus, the results might not be necessarily universal for an old population who had secondary osteoporosis. Participants with missing data have also been excluded in several analyses. This is a cross-sectional study that only reveals the association between muscle/fat and bone, but not the causal relationship. Prospective longitudinal or interventional studies are warranted in the future.

In conclusion, skeletal muscle mass was positively related to bone structures, while body fat percentage and age were negatively associated with bone health. BMD was non-linearly associated with increased BMI, of which the increase rate generally slowed down in bearing bones. Increased ASMI did not slow down BMD increase rate in lumbar spine and total hip as BMI and FMI, especially in male subjects. BMI-defined obesity failed to significantly reduce the risk of osteoporosis in the adjusted model when the reference group involved people with normal BMI and without sarcopenia. BF%-defined obesity was not associated with improved bone health. Sarcopenia was significantly related to increased risk of osteoporosis. Old people with higher body weight, better lower extremity performance, and larger limb lean mass had lower prevalence of osteoporosis. The improvement of muscle status should be regarded as a clinical recommendation for elderly people to prevent age-related osteoporosis.

## Data availability statement

The original contributions presented in the study are included in the article/**Supplementary Materials**, requests to access these datasets should be directed to Ling Qin, lingqin@cuhk.edu.hk.

## Ethics statement

The studies involving human participants were reviewed and approved by The Joint Chinese University of Hong Kong – New Territories East Cluster Clinical Research Ethics Committee (Affiliation: The Chinese University of Hong Kong and New Territories East Cluster). The patients/participants provided their written informed consent to participate in this study.

## Author contributions

CL: writing—original draft and editing, conceptualization, and methodology. PW: methodology and writing—review and editing. XT: statistical analysis and data visualization. SC: supervision and writing—review and editing. VH: investigation and data curation. W-HC: supervision and writing—review and editing. QL: investigation, supervision, and writing—review and editing. SL: conceptualization and validation. RW: conceptualization, investigation, supervision, and writing—review and editing. All authors contributed to the article and approved the submitted version.
