# Supplementary material for: Muscle plays a more superior role than fat in bone homeostasis: A cross-sectional study of old Asian people
Source: Front Endocrinol (Lausanne). 2023 Jan 12;13:990442. doi: 10.3389/fendo.2022.990442 (PMC9877339; doi:10.3389/fendo.2022.990442)
Supplement: Supplementary file 1 [file Table_1.docx]

Supplementary Material

# Supplementary Table S1. The description of the bone and body composition parameters.

| **Abbreviation** | | **Parameters** | **Description** | **Unit** |
| --- | --- | --- | --- | --- |
| **Density parameters** | | | | |
| aBMD | Areal bone mineral density | | Amount of bone mineral divided by the surface area of the bone | g/cm^2^ |
| Tot.vBMD | Toal volumetric bone mineral density | | Bone mineral mass divided by the bone volume | mg HA/cm^3^ |
| Ct.vBMD | Volumetric cortical bone mineral density | | Volumetric density in cortical bone | mg HA/cm^3^ |
| Tb.vBMD | Trabecular volumetric bone mineral density | | Volumetric density in trabecular bone | mg HA/cm^3^ |
| **Structural parameters** | | | | |
| BV/TV | Trabecular bone volume fraction (Bone volume / Total volume) | | Ratio of the segmented trabecular bone volume to the total volume | % |
| Tb.Th | Trabecular thickness | | Mean thickness of the trabeculae | mm |
| Ct.Th | Cortical thickness | | Calculated as the mean cortical volume divided by the outer bone surface | mm |
| Stiffness | N/A | | Estimate of bone strength by a uniaxial compression test | kN/mm |
| **Body composition parameters** | | | | |
| ASMI | Appendicular skeletal muscle index | | Muscle mass from arms and legs in kilograms divided by height in meters squared | kg/m^2^ |
| FMI | Fat mass index | | Total fat mass in kilograms divided by height in meters squared | kg/m^2^ |
| TL fat ratio | Trunk-to-limb fat ratio | | Ratio of trunk fat to limb fat | N/A |
| Limb LTF ratio | Limb lean-to-fat ratio | | Ratio of lean mass to fat mass in four limbs | N/A |
| Total LTF ratio | Total lean-to-fat ratio | | Ratio of lean mass to fat mass in the whole body | N/A |
